# Animal Welfare‐Friendly Noninvasive Oral Fluid in Swine Diagnostics: Current Applications in Disease Monitoring, Key Challenges and Future Directions

**DOI:** 10.1155/vmi/5524629

**Published:** 2026-08-05

**Authors:** Chandra Shaker Chouhan, Flaminia Coiacetto, Sam Abraham, Jasim Muhammad Uddin

**Affiliations:** ^1^ School of Veterinary Medicine, Murdoch University, Perth Western Australia, 6150, Australia, murdoch.edu.au; ^2^ Department of Medicine, Faculty of Veterinary Science, Bangladesh Agricultural University, Mymensingh 2202, Bangladesh, bau.edu.bd; ^3^ School of Medical, Molecular and Forensic Sciences, Murdoch University, Perth Western Australia, 6150, Australia, murdoch.edu.au; ^4^ Centre for Biosecurity and One Health, Harry Butler Institute, Murdoch University, Perth Western Australia, 6150, Australia, murdoch.edu.au; ^5^ Centre for Animal Production and Health, Food Future Institute, Murdoch University, Perth Western Australia, 6150, Australia, murdoch.edu.au

**Keywords:** animal welfare-friendly noninvasive sampling, herd health surveillance, oral fluid diagnostics, swine biomarkers, swine pathogens

## Abstract

The integration of oral fluid (OF) sampling into veterinary diagnostics represents a significant shift in animal herd health monitoring, offering a noninvasive alternative to traditional blood‐based methods. OF, predominantly composed of saliva, mucosal transudate and plasma‐derived components, contains immunoglobulins, acute‐phase proteins and nucleic acids that mirror both local and systemic physiological processes. These components enable a thorough evaluation of immune responses and inflammatory status, as well as the detection of economically significant infectious pathogens in swine. OF can be collected noninvasively using cotton ropes or swabs, which is feasible for individual or pen‐level herd health sampling with minimal animal handling and fosters an animal‐friendly approach. Several studies have demonstrated pathogen antigen detection, particularly PCR‐based detection of shedding pathogens, as well as antibody and biomarker measurements in OF; however, concentrations are often lower than those observed in other biological fluids, especially in serum. In addition, diagnostic interpretation is further constrained by poor pig participation, rope chewing due to dominance, low prevalence within pens, contamination with feed or environmental material, instability of analytes during transport and storage, and the scarcity of validated assays specific to OF. This review summarises OF production, composition and diagnostic analyte entry pathways from blood to OF and current applications for detecting pathogens and biomarkers in pigs and critically examines methodological factors that influence diagnostic performance, including sample collection procedures, handling and assay optimisation, such as dilution and threshold selection. Finally, it outlines priorities for future development, including standardised OF collection and handling protocols, OF‐validated pathogen and biomarker assays, matrix‐specific thresholds, regulatory validation and proteomic biomarker discovery to enable earlier detection and support continuous herd health monitoring in global swine production.

## 1. Introduction

The emergence of oral fluid (OF) as a diagnostic sample represents a significant advancement in veterinary diagnostics, offering new avenues for welfare‐friendly health monitoring in pigs [1]. This fluid, primarily composed of saliva, mucosal transudate and plasma‐derived components, serves as an essential lubricant, helps combat microbes, aids digestion and enhances immune defences [2]. OF has a spectrum of diagnostic biomarkers, including immunoglobulins (IgA, IgG and IgM), acute‐phase proteins, nucleic acids, hormones, enzymes, electrolytes and metabolites, which are both local and systemic contributions [1, 3]. These biomarkers provide a holistic view of local and systemic health indicators, enabling the assessment of immune competency, efficacy of immunisation and prompt detection of pathogenic exposure [4].

Traditionally, blood sampling is a staple of diagnostic endeavours; however, blood collection is time‐consuming and invasive, causing significant stress in animals [5]. In pigs, particularly boars, sows and group‐housed animals, blood collection can be labour‐intensive and difficult, may pose safety risks to handlers and can cause complications such as needle breakage and post–blood collection haematoma formation [6]. Owing to its invasiveness, it often restricts recurrent sampling, compromises animal numbers and precludes continuous herd‐level monitoring. In contrast, OF sampling is easy and nonstressful, requiring minimal restraint and training. This method facilitates pen‐level or individual repeated sampling, which is favourable for routine surveillance, longitudinal studies, early outbreak detection and herd health monitoring [7–9].

OF sampling is widely used in human medical diagnostics to detect antigens and antibodies against a range of pathogens [10–12]. The promising results achieved in this area have led to its translation into the field of veterinary medicine. OF sampling is widely used to monitor the health of pig herds in leading pork‐producing nations. Importantly, OF has identified several major porcine pathogens, including porcine circovirus (PCV), porcine reproductive and respiratory syndrome virus (PRRSV), porcine epidemic diarrhoea virus (PEDV), African swine fever virus (ASFV), influenza A virus (IAV), classical swine fever virus (CSFV), foot‐and‐mouth disease virus (FMDV) and *Actinobacillus pleuropneumoniae* (APP) [1, 13, 14].

OF is beneficial; however, it has lower antibody levels than serum, which impairs its diagnostic accuracy. Analytical challenges also arise from the dilution factor and the absence of OF‐specific diagnostic kits. However, uniformity in sample collection and handling, assay optimisation and the development of OF‐based test kits are improving the diagnostic value of OF [4]. These improvements are progressively surpassing existing constraints and support the broader use of OF as a practical tool for large‐scale disease surveillance and diagnostics in swine.

Earlier reviews have explored the detection of viral pathogens in pigs utilising OF, along with aspects of animal welfare and biomarker [1, 2, 15–17]. There is a paucity of comprehensive reviews that encompass both the analyte entry pathways of OF and the practical diagnostic challenges associated with OF sampling. This review eliminates this gap by synthesising the existing evidence on OF‐based diagnostics in pigs. This review specifically aims to (i) summarise the production and composition of OF pertinent to diagnostics; (ii) synthesise evidence for OF‐based detection of significant swine pathogens and biomarkers; (iii) identify principal challenges impacting diagnostic accuracy and reproducibility; and (iv) delineate practical recommendations and future research priorities to enhance the usage of OF in welfare‐friendly herd health surveillance.

## 2. Search Strategy and Selection Criteria

This narrative review with critical synthesis was based on targeted searches across PubMed/Medline, Google Scholar, Web of Science and Scopus for studies on swine OF diagnostics. The search covered English‐language literature published up to 2024. The search employed a combination of terms such as ‘oral’ fluid ‘OF’, ‘saliva’, ‘swine’, ‘pig’, ‘diagnostics’, ‘surveillance’, ‘qPCR/RT‐qPCR’, ‘ELISA’, ‘biomarkers’ and ‘pathogen’. Original experimental and field research was included if it involved pathogen detection, antibody identification, biomarker measurement, sampling methods, assay performance or herd‐level surveillance using swine OF or saliva. Studies that detected pathogens using OF or saliva and compared these samples with other diagnostic matrices, including serum, nasal swabs, faeces, tissue, tracheal swabs or other biological samples, were prioritised for inclusion in Table 1. Articles were excluded if they did not pertain to swine, did not involve OF or saliva or did not provide relevant information on diagnostic testing, biomarker assessment or disease surveillance. Table 1 was constructed from eligible primary studies by extracting key diagnostic information, including pathogen, analyte and test method, OF collection and storage conditions, dilution strategy, comparator matrix, relative performance of OF and the best use of OF in swine disease In addition, pertinent review articles and background literature were searched to support the general sections on the composition, function, production and mechanisms of OF.

**TABLE 1:** Swine pathogen monitoring via oral fluid: analyte and test methods, collection and storage conditions, dilution/extraction protocols, comparator, relative performance and best‐use applications.

Pathogen	Analyte & test method	OF collection and storage	Dilution/extraction protocols	Comparator	Relative performance of OF	Best use of OF	Ref.
PRRSV	PRRSV RNA by RT‐qPCR; PRRSV IgG/IgA by ELISA/IFAT	5/8″ cotton rope, 20–30‐min chew, −20°C	Undiluted for RT‐qPCR; 1:2 for antibody assays	Serum; buccal swabs	Serum RT‐qPCR detected PRRSV for ∼5 weeks and OF for ∼4 weeks. RNA kinetics were similar, but OF had a lower viral load (*R* ^2^ = 0.56). Buccal swabs performed poorly. The RT‐qPCR sensitivity was ∼81–88% during the first 4 weeks; OF IgG was low, and IgA was inconsistent.	Repeated pen‐/herd‐level PRRSV surveillance; not a direct replacement for individual serum viremia or serology.	[7]
PRRSV	PRRSV IgG by ELISA	Individual swab/pen rope (15 pigs/rope), 45‐min chew, −20°C	ELISA diluted per manufacturer; S/P ≥ 0.4	Individual/pooled serum	OF‐ELISA Se was serum‐comparable (∼96% finishers; ∼95% sows), with pooled OF/serum Sp > 95%. Pen OF agreed well with serum in finishers (*κ* = 0.88) and individual OF (*κ* = 0.81), but failed in ∼40% of grouped‐housed sow pens; individual sow OF had a lower Sp	Economical PRRSV antibody surveillance; however, poor participation, dominance and aggression may limit OF sampling reliability.	[18]
PRRSV	PRRSV RNA by RT‐qPCR; PRRSV IgG by ELISA	Cotton/polyester rope; length (L): 1 m, diameter (D): 14 mm, 30‐min chew, −80 °C	Undiluted for RT‐qPCR; ELISA diluted per manufacturer; S/P ≥ 0.4	Serum	Serum was more RNA‐positive early, up to ∼5 dpi; serum and OF were similar at 7–21 dpi; OF was higher from 28 dpi. Serum‐OF‐ELISA agreement was excellent.	Serum is preferable for early PRRSV detection, whereas OF is useful for later/prolonged RNA detection and IgG monitoring.	[19]
PRRSV	PRRSV RNA by RT‐qPCR; PRRSV IgG by ELISA	Cotton/polyester rope; L: 1 m, D: 14 mm, 30‐min chew, −80 °C	150 μL OF for extraction; ELISA diluted per manufacturer; S/P ≥ 0.40	Individual Serum	OF RT‐qPCR specificity was > 90%; detection declined with age/low prevalence, but OF was always PCR‐positive when ≥ 30% of pigs in a pen were serum‐positive. OF‐ELISA Se was 100% and Sp 82%.	Field pen‐level PRRSV monitoring; useful when within‐pen prevalence is moderate/high, but may miss older or low‐prevalence pens.	[20]
PRRSV	PRRSV IgG/IgM/IgA by modified ELISA	Pen rope, 20–30‐min chew, −80°C	1:2	Serum	IgG was detected at useful levels in field and experimental OF up to 126 days; IgG Se 94.7% and Sp 100%; IgM/IgA were mainly useful in synchronised experimental infection.	Cost‐effective PRRSV herd monitoring and elimination program; IgG is preferred over IgM/IgA for field OF testing	[21]
PRV	PRV‐IgG by indirect ELISA	1.27‐cm 3‐strand rope, 30–45 min chew‐20°C	1:1	Serum	OF IgG followed serum trends but was lower; AUC 0.93, ICC 99.3%, adjusted *R* ^2^ = 0.6503. OF Sp was high (99.2%–100%), but Se was cut‐off dependent	PRV population surveillance and DIVA‐compatible OF‐ELISA development; serum remains stronger for individual serology.	[14]
PEDV	PEDV RNA by RT‐qPCR; PEDV‐specific IgA/IgG by ELISA	3‐strand rope, 20–30‐min chew	Undiluted for RT‐qPCR; 1:2 for antibody assays	Rectal swabs, pen faeces; serum	PEDV RNA detected in all sample types at 6 DPE; OF and rectal swabs remained positive to 69 DPE, faeces to 55 DPE. OF was equal/better than rectal swabs and pen faeces; OF IgA ELISA Se/Sp 1.00/1.00, OF IgG 0.69/0.97.	Pen‐level PEDV RNA surveillance and mucosal IgA monitoring; faeces/rectal swabs remain relevant for enteric shedding confirmation.	[22]
FMDV	FMDV RNA by RT‐qPCR; live virus by LFBKαvβ6 cells; FMDV antigen by DAS‐ELISA/LFIST; FMDV‐O IgA by ELISA	5/8″ cotton rope, 20–30 min chew, −80°C	Undiluted for RT‐qPCR/VI/antigen tests; 50 μL for LFIST; 1:2 for IgA ELISA	Oral/nasal swabs; serum	OF RNA detected 1–21 dpi, earlier/prolonged than swabs/serum; live virus 1–5 dpi; LFIST antigen up to 6 dpi; DAS‐ELISA 2–3 dpi; OF IgA from 14 dpi with 100% Se/Sp	Early FMDV surveillance before clinical signs under experimental conditions; field validation is needed before the routine replacement of official specimens.	[23]
PRRSV/PCV2/HEV	PRRSV RNA by RT‐PCR; PCV2 DNA by PCR; HEV RNA by RT‐PCR PCV2 DNA by PCR HEV RNA by RT‐PCR	3‐strand rope, 30‐min chew −70°C	140 μL OF supernatant used for extraction;	Faeces; serum	PRRSV was detected in OF from 7 to 11‐week‐old weaners and fatteners; fattener serum was negative. PCV2 was detected in OF from weaners and was more prevalent than in faeces, but not fully serum‐concordant. HEV was detected in OF/faeces from 5–9‐week‐old weaners, while serum positivity was low.	Group‐level screening for PRRSV, PCV2 and HEV; serum remains useful for confirming viremia, especially for PCV2.	[24]
PCV2	PCV2 DNA by qPCR	Cotton rope, 30 min chew, −80°C	1:10	Serum pools	OF detected more positive pens than serum pools and had higher viral loads by ∼1.8–2.1 log10 copies/mL; serum–OF correlation was inconsistent.	Good for presence/absence, less reliable for estimating exact pig‐level viral load.	[25]
PCV2	PCV2 DNA by qPCR; PCV2 IgG/IgA/IgM by ELISA	5/8″ 3‐strand rope, 20‐min chew, −80°C	Undiluted OF for extraction, OF 1:3 for IgG/IgA/IgM ELISA.	N/A	OF qPCR detected PCV2 from 2 to 98 dpi with Se/Sp 98%/100%; OF IgG/IgA/IgM appeared from ∼14 dpi, and IgG/IgA persisted to 98 dpi.	Pen‐level longitudinal monitoring of PCV2 shedding and antibody response.	[26]
PCV2	PCV2 DNA by qPCR	Pen rope/swab, −20°C	200 μL OF used for extraction	Serum/faecal pools	OF detected PCV2 circulation, but serum/faecal viral loads better reflected vaccination status; OF prevalence did not distinguish vaccinated from nonvaccinated farms.	Routine PCV2 surveillance; interpret cautiously due to possible environmental contamination and use serum for vaccine‐failure assessment.	[27]
PCV3	PCV3 DNA by PCR	Cotton rope (L: 1.2 m, D: 1.5 cm), 30–40‐min chew, −80°C	Undiluted OF supernatant for extraction	Stall‐based vs pen‐based OF	PCV3 positivity was 12.3%; positivity was higher in stall‐based sow OF samples (15.06%) than in pen‐based commercial‐pig OF samples (9.21%). Codetection with PCV2 occurred in 41% of PCV3‐positive samples, and PCV3 complete genomes and cap genes were sequenced for phylogenetic analysis.	Broad, noninvasive herd screening and molecular epidemiology.	[28]
ASFV/CSFV/FMDV	ASFV DNA; CSFV/FMDV RNA by multiplex RT‐qPCR	Rope, −70°C	250 μL OF used for nucleic acid extraction	Oral swabs	ASFV was detected at 3 dpi before severe signs, CSFV at 5 dpi with clinical onset and FMDV at 1 dpi before signs.	Rapid transboundary‐disease surveillance; experimental findings require field validation and threshold studies.	[29]
JEV	JEV RNA by RT‐qPCR	1.25‐cm twisted rope, −80°C	Centrifuged OF was used directly for extraction	N/A	JEV RNA was detected in OF during acute infection: domestic pigs 3–14 dpi, peak 5 dpi; miniature swine mainly 2–7 dpi, with NS5 assay 2 most sensitive.	Experimental JEV oral‐shedding herd surveillance; field validation is needed before routine use.	[30]
SVDV	SVDV RNA by RT‐qPCR; VI by LFBKαvβ6; SVDV antibodies by C‐ELISA; SVDV IgM/IgA by indirect ELISA	5/8″ cotton rope, 20–30 min chew, −80°C	Centrifuged OF used for extraction; C‐ELISA from 1:2; IgM/IgA ELISA at 1:10.	Serum; oral swabs; nasal swabs;	OF RNA detected 1–21 dpi and was stronger/prolonged than swabs/serum; VI positive mainly 1–5/7 dpi; IgM Se/Sp 91.7%/100%, IgA Se/Sp 100%/100%.	Vesicular disease surveillance, including subclinical infection; antibody interpretation is time‐dependent and routine replacement needs further validation.	[31]
IAV	IAV RNA by RT‐qPCR; IAV antigen by POC assay; live IAV by MDCK cells	1.3‐cm pen‐based cotton rope, −80°C	OF was used directly for extraction	Nasal swabs	OF RT‐qPCR showed the highest detection probability for most DPIs; OF and nasal swabs were similar early, but OF was better after DPI 6. VI was better from nasal swabs than OF	Pen‐level IAV RT‐qPCR surveillance; NS preferred for VI/individual diagnosis.	[32]
IAV	IAV RNA by RT‐qPCR	5/8″ 3‐strand cotton rope, 20–30‐min chew, −20°C	OF supernatant used for extraction	NS	OF Se 80.4%, Sp 100%, *κ* = 0.82; detection 99% at ≥ 18% within‐pen prevalence, 69% at 9%, VI positive in 51% of PCR‐positive OF	Pen‐level influenza surveillance: reduced reliability at very low prevalence	[5]
MHP	MHP DNA by qPCR	1.6 cm 3‐strand rope, 25 min chew, −80°C	OF was used directly for extraction	Tracheal swabs	OF (3–12 dpi) than tracheal swabs (3 dpi); detection rose with increasing within‐pen prevalence (95.7% at 100% prevalence).	Pen‐level MHP surveillance is limited to moderate‐ to high‐prevalence settings; for low‐prevalence settings or individual diagnosis, consider other approaches.	[33]
APP	APP IgA by ELISA	Salivation induced with azaperone or ascorbic acid swab, −20°C	Twofold dilution in PBST	Serum	Salivary IgA was detectable early after infection, whereas serum antibodies were low/absent and declined later, whereas serum antibodies persisted.	Early mucosal antibody detection is not reliable alone for late‐stage infection surveillance.	[34]
APP	ApxIV IgM/IgA/IgG by ApxIVAb ELISA	1.27‐cm 3‐strand cotton rope, 30‐min chew, −80 °C	1:2	Serum	OF ApxIV antibodies were detectable mainly in Serovars 1 and 7; OF IgG response appeared after serum response and was minimal for Serovars 5 and 12	Population monitoring of APP exposure using the OF ApxIV antibody; useful experimentally, but OF cut‐offs and field validation are needed.	[13]
Enterotoxigenic *E.coli* (ETEC) K88	K88‐specific IgA by ELISA	Saliva collected on Day 15	Not reported	Plasma, faeces and jejunal tissue	K88‐specific IgA was high in nonmedicated fish‐protein pigs but low in spray‐dried plasma/medication.	Mucosal immune biomarker for ETEC challenge studies; not a standalone diagnostic test.	[35]
*Streptococcus suis*	*S. suis* Serotype 9 by Culture/PCR	Saliva swab, −20°C	10‐fold in saline	Tonsil brushing	Saliva broadly agreed with tonsil brushing for detecting colonisation/shedding.	Noninvasive monitoring of *S. suis* shedding and colonisation, but not a standalone diagnostic test.	[36]
*Helicobacter spp.*	Helicobacter sp. DNA by PCR	Saliva swab, −80°C	Used directly for extraction	Gastric tissue and faeces	Saliva was positive for *Helicobacter* spp. in 64% of samples, similar to faeces (60%); however, both matrices were negative for *H. suis* and *H. pylori* by species‐specific PCR.	*Helicobacter* spp. Screening only; gastric tissue is required for *confirmation of H. suis*.	[37]

## 3. Composition, Function and Production of OF

**FIGURE 1:**
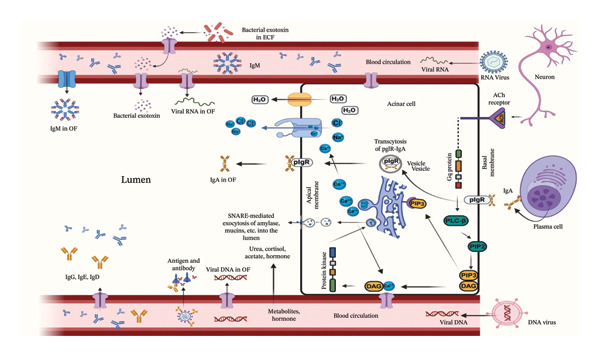
Schematic representation of immune components and biomarker transfer in OF.

OF is a dynamic biological fluid primarily composed of saliva, which is regulated by the autonomic stimulation of the salivary glands (Figure 1) [38, 39]. It is secreted mainly by the three major salivary glands: the parotid, submandibular and sublingual. The parotid glands release enzyme‐rich serous fluid, whereas the submandibular and sublingual glands produce seromucous secretions containing mucins and proteins [40]. Additional contributions come from the minor salivary glands and gingival crevicular fluid [1]. OF also contains other components, including mucosal and plasma transudates, inflammatory mediators, respiratory secretions, epithelial cells, feed residues, bacteria and viruses [5]. Beyond glandular sources, OF incorporates substances from the circulatory and lymphatic systems via passive diffusion, ultrafiltration and active transport across the semipermeable oral mucosa [41]. This allows for the exchange of small molecules, such as water (over 99%), ions, urea and hormones; capillary transudates associated with the mucosa; and larger compounds, such as antibodies (IgG, IgM), nucleic acids (DNA/RNA), bacterial exotoxins, hormones (cortisol), proteins and lipids via receptor‐mediated transport through capillaries [14]. Inorganic components, such as bicarbonate, phosphate and potassium, are transported through salivary ducts [14]. OF comprises mucosal immunoglobulin A (IgA), antimicrobial proteins, mucins and enzymes such as amylase and is additionally augmented by environmental inputs, including microbes and feed particles [42]. From a physiological point of view, OF is central in digestion, lubrication and immunological defence. The digestive cascade is initiated by alpha‐amylase, which hydrolyses carbohydrates and moistens food [41]. Mucins create a viscoelastic coat on the oral epithelium, lubricate it and protect mucosal surfaces from desiccation. Buffering capacity and oral pH were maintained using bicarbonate and phosphate ions, respectively. Antimicrobial proteins and secretory IgA in OF modulate the oral microbial ecology.

## 4. OF Collection, Handling and Storage

**FIGURE 2:**
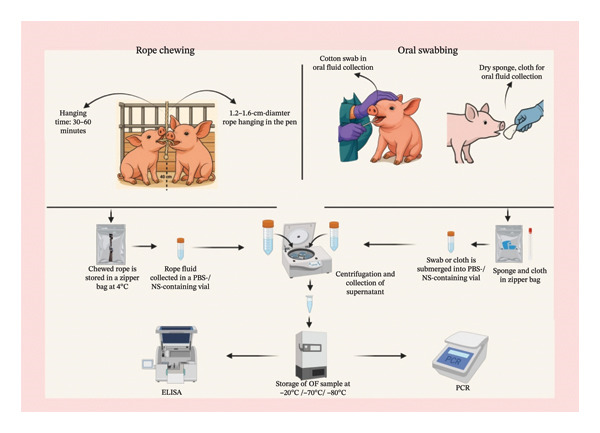
Workflow for oral fluid collection, handling and storage.

The use of OF in veterinary diagnostics is increasingly being adopted owing to its noninvasive sampling, improved animal welfare and numerous diagnostic analyses that can be performed on it. Factors that can impact sampling and, therefore, the results of diagnostic tests include the animal’s age, behaviour, group size, environmental enrichment, dominance status, slatted or straw‐bedded housing, collection time of day, number of ropes, familiarity with the collection rope and duration of sampling [2, 43]. Collection is typically more effective in the early morning before feeding, when pigs are more active, and OF is naturally higher. The OF collection can be either stimulated or unstimulated; stimulated OF is preferred because of its higher volume and lower viscosity, which eases laboratory processing [16]. In pigs, mechanical stimulation using hanging cotton ropes or sponges is common because they have an instinctive tendency to explore and chew on unfamiliar objects [2]. Rope‐based OF collection is usually used for weaned piglets and grower pigs, where three‐strand cotton ropes (1.6 cm diameter for grower/finish and adult pigs; 1.2 cm for nursery pigs) are suspended at shoulder height (∼40 cm above the floor) in each pen to promote chewing **(**Figure 2) [14, 44]. Contaminated ropes have the potential to introduce extraneous materials or pathogen nucleic acids into OF samples, particularly those excreted in faeces or those persisting in the pen environment. Therefore, ropes should be positioned away from pen walls, feeders, waterers, floors, faeces, bedding, feed residues and standing water and should be hung from gates, rafters or brackets to facilitate access without entering the pen, allow multiple pigs to interact simultaneously and minimise environmental contamination. [2]. In the majority of experimental studies, one rope was used per 15–25 pigs [4, 14, 18, 45]. Sampling durations are typically reported as 20–30 min, although some studies have noted shorter (e.g., 15 min) or longer (e.g., 45–60 min) durations, allowing sufficient interaction [14, 43]. Sampling requirements under Wisconsin regulations mandate one OF sample for farms with fewer than 150 pigs and three samples for larger herds [44]. Training pigs by initially placing ropes on the floor before suspending them, along with increasing the number of ropes, can enhance participation and increase the average chewing time per pig, while flavour additives (e.g., fruit juice) may increase suckling pig interest but with variable effects [2]. For piglets that are preweaning or approaching weaning, OF can be collected through litter oral fluid (LOF) or family oral fluid (FOF) sampling. This involves providing a cotton rope to either the piglets alone or both the sow and the piglets [46, 47]. In situations where rope‐based collection is impractical, oral swabs may be obtained from weanling piglets and subsequently placed in phosphate‐buffered saline (PBS) for molecular analysis [48]. However, poor participation and dominance‐related chewing behaviour may compromise the representativeness of pen‐based OF samples, as dominant or more inquisitive pigs may contribute disproportionately, whereas sick pigs may contribute minimally or not at all. Therefore, inadequate rope interaction should be considered a potential source of sampling bias. Individual sponge‐ or cloth‐swab sampling may be preferable when reliable pen‐level rope interaction is not achieved, particularly in sick or older pigs, sows, gilts, boars, solitary housed animals or animals with low curiosity [15, 43]. Sponges or dry swabs of cloth were held with forceps and offered to the pigs until they were thoroughly moistened with OF. Following collection, OF samples must be handled appropriately to prevent degradation, whether by using a rope a sponge‐dry swab cloth**.** OF from ropes/sponges or dry swab cloth is usually wrapped into a zipper (Ziploc) bag**,** and the bottom corner of the bag is cut; the fluid is then transferred into sterile tubes. All samples should be centrifuged promptly (3000 × g, 10 min, 4°C) to remove mucus, food particles, epithelial cells and other contaminants, which helps improve sample clarity and reduces enzymatic activity that can degrade proteins and biomarkers [14]. During the shipment of the samples, it was imperative to maintain them in a cool ice box. Samples can be stored at 4°C for up to 48 h for short‐term preservation. For long‐term storage, it is essential to freeze samples at −20°C, preferably at −80°C, particularly when dealing with enzymes, proteins or biomarkers prone to degradation [16, 41].

## 5. Potential Applications of OF in Swine Diagnostics

### 5.1. Detection of Swine Biomarkers in OF

**FIGURE 3:**
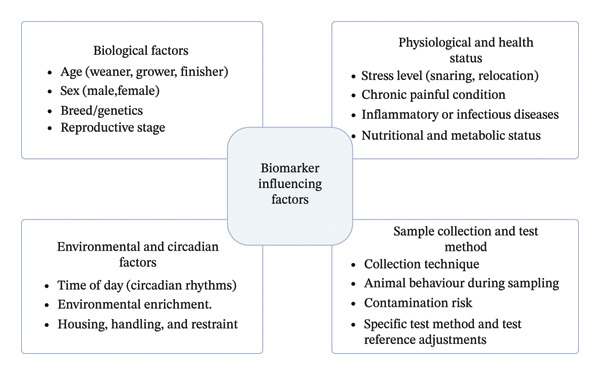
Key biological, environmental and methodological factors influencing OF biomarker.

Pig OF is a significant resource for monitoring stress, inflammation, immunological status and disease, with many biomarkers showing strong correlations with serum levels because OF contains approximately 30% of blood biomolecules. [2]. Porcine OF is rich in biomarkers, including acute‐phase proteins, stress markers, enzymes, metabolites, hormones and nucleic acids [2, 15]. The most prevalent APPs include pig major acute‐phase protein (Pig‐MAP), haptoglobin (Hp), serum amyloid A (SAA), C‐reactive protein (CRP) and alpha‐1‐acid glycoprotein (AGP) [49]. Although their concentrations in OF are physiologically lower than those in the blood, they rise during inflammation or infection, with mild to strong correlations observed between OF and serum levels in previous studies [15, 50]. Stress biomarkers such as cortisol, chromogranin A (CgA) and salivary alpha‐amylase (sAA) can be reliably measured in OF. Cortisol remains the gold standard for assessing hypothalamic–pituitary–adrenal (HPA) axis activation, which is increased under stressors such as transport and restraint and is detected in pigs [6, 51]. CgA and sAA are responsive to both acute and chronic stress, indicating functioning of the autonomic nervous system (ANS) [15]. Markers of oxidative stress, including hydrogen peroxide (H_2_O_2_), advanced oxidation protein products (AOPPs) and total antioxidant capacity (TAC), play a vital role in assessing redox homeostasis in pigs, particularly before farrowing or during disease exposure [15]. Enzymatic biomarkers, including adenosine deaminase (ADA), which increases in respiratory and inflammatory conditions, alongside butyrylcholinesterase (BChE), total esterase activity (TEA) and salivary lipase (sLip), surge in pigs in pain [52]. The functions of carbonic anhydrase VI (CA‐VI), which has been detected in pigs, include the modulation of immune responses and pH homeostasis [17, 53]. Corticosterone and testosterone levels, which correlate with reproductive status, stress and behaviour, have been measured in the OF of pigs [15]. Metabolites, such as butyrate and 2‐hydroxyvalerate, are markers of the preoestrus stage in sows, aiding oestrus detection and improving reproductive efficiency [2]. Copper and zinc are essential nutrient elements that can also be measured in the OF of pigs [50]. Recent proteomic studies using OF have characterised a set of structural proteins, such as lipocalins, members of the S100 protein family, and y‐ and albumin‐derived proteins, odorant‐binding protein, chitinase, lipocalin‐1 and alpha‐2‐HS‐glycoprotein, whose expression profiles differ depending on the health status and sex of the pigs, thus emphasising the need to establish sex‐specific reference ranges [16, 51, 53, 54]. Although there is a correlation between many OF biomarkers and those found in serum, OF should not be considered a direct substitute for blood. This is because several analytes are present at lower, more variable concentrations in OF. OF is particularly beneficial for repeated, noninvasive monitoring of stress, welfare, mucosal responses, redox status and locally secreted biomarkers, as it avoids venipuncture and strong restraint. On the other hand, serum is preferred when precise systemic biomarker quantification, validated clinical cut‐offs and individual‐level interpretation are required. Therefore, OF biomarker results should be interpreted with caution, especially for low‐abundance analytes, pooled pen‐level samples or conditions affecting only a small proportion of pigs, where the biomarker signal may be diluted or underestimated [15, 50]. As illustrated in Figure 3, the interpretation of OF biomarkers is affected by a range of factors, including biological, physiological, environmental, circadian, collection‐related and assay‐related influences (Figure 3). Factors such as age, sex, breed and genetic factors, reproductive status, and production stage—including the weaner, grower, and finisher stages—may influence OF biomarkers in swine, including cortisol, ADA, CRP, Hp, and estrus‐related proteins [8, 9, 15]. Acute stress induced by invasive sampling and animal displacement increases cortisol, salivary alpha‐enzymes and CgA. In contrast, chronic conditions, such as lameness and perinatal dysregulation, modify the activities of BChE, TEA and CgA [15, 52]. Inflammatory or infectious diseases trigger the production of APPs, such as SAA, Hp, CRP and Pig‐MAP, as well as enzymes such as ADA, in pigs [15, 52, 55]. Environmental factors, including time of day, enrichment, housing and handling, impact biomarker levels, notably cortisol and CgA [15, 52]. Factors such as storage conditions during transport and possible contamination from feed residues, faeces, bedding materials and pen surfaces, as well as assay‐related factors such as dilution factors, significantly impact the reliability of biomarker measurements in swine [14, 15, 50]. A range of analytical serum‐based kits originally designed for human and veterinary diagnostics have been co‐opted for the quantification of biomarkers in swine OF. Immunoassays, such as enzyme‐linked immunosorbent assay (ELISA), time‐resolved fluoroimmunoassay (TR‐FIA) and radioimmunoassay (RIA), are used to detect cortisol, Hp, SAA, CRP, CgA and oxytocin, and total proteins through the Bradford assay and microplate titration for TAC in swine [2, 15]. Enzymatic assays, including colorimetric, spectrophotometric and fluorometric methods, were used to detect ADA, TEA, sAA and BChE. Advanced proteomic techniques, such as two‐dimensional gel electrophoresis (2‐DE) with matrix‐assisted laser desorption/ionisation‐time of flight (MALDI‐TOF), isobaric tag for relative and absolute quantification (iTRAQ) and liquid chromatography–mass spectrometry (LC–MS), enable the detailed analysis of proteins and peptides in OF in pigs [2, 15, 51, 53].

### 5.2. Detection of Swine Pathogens in OF

OF is increasingly recognised as a reliable and adaptable diagnostic matrix for detecting bacterial and viral infections in pigs. This review highlights economically significant pig pathogens and describes their OF collection methods, analytes, diagnostic protocols, dilution or extraction conditions, comparator matrices, relative diagnostic performance and best‐use applications (Table 1). We also examined how these factors affect the diagnostic efficacy, even when similar pathogens are detected using different sampling and testing approaches**.** OF facilitates the detection of nucleic acids through polymerase chain reaction (PCR), quantitative PCR (qPCR), reverse‐transcription qPCR (RT‐qPCR) and whole‐genome sequencing (WGS). This method often allows earlier or more sustained detection than serum, nasal swabs or other comparator matrices, contingent on the pathogen and the stage of infection [1, 5, 23]. Optimising test protocols, especially sample processing, dilution or extraction volume, assay cut‐off and cycle threshold (Ct) values, are often required to improve both sensitivity and specificity. Major pathogens detectable through OF include PRRSV, PEDV, FMDV (1 day postinfection), ASFV (3 days postinfection), CSFV, PCV2 (2 days postinfection), *Mycoplasma hyopneumoniae* (MHP), Japanese encephalitis virus (JEV), IAV and swine vesicular disease virus (SVDV), APP, *Streptococcus suis*, enterotoxigenic *E.coli* (ETEC) K88 and *Helicobacter* spp**.** [23–25, 29, 31]. It is important to note that the diagnostic performance of OF is not uniform across pathogens or assay types. In the context of PCR‐based detection, OF is particularly advantageous for surveillance at the pen or herd level to detect pathogen shedding. However, its sensitivity depends on factors such as sampling timing, prevalence within the pen, extraction method, assay protocol and Ct value. In pens or herds with low prevalence, OF may provide false‐negative results, as only a small fraction of infected pigs may contribute pathogen or antibody to the pooled sample. For instance, serum is preferred for early detection of PRRSV viremia and individual serology; nasal swabs are favoured for individual IAV diagnosis and virus isolation (VI); tracheal swabs exhibit greater sensitivity for early MHP detection; and gastric tissue is necessary for definitive confirmation of *H. suis*. In terms of antibody detection, OF‐based ELISAs generally showed a significant correlation with standard serum assays; however, parameters such as sample dilution, use of undiluted samples, 1:2 or 1:3 dilution, assay incubation time and cut‐off thresholds often require matrix‐specific optimisation, especially for new pathogenic agents. OF samples are suitable for detecting the IgG, IgA and IgM isotypes [7, 13, 22]. IgG is strongly correlated with serum levels in infections caused by PRRSV, PCV2 and APP [7, 13, 26]. IgA, particularly in PEDV and APP, reflects early mucosal immune responses, whereas IgM indicates acute‐phase activity, but may vary under field conditions [22]. The use of OF is also suitable for VI in cell cultures, including FMDV, SVDV and IAV, where high titres and early infectivity have been reported in pigs [23, 31]. Nevertheless, VI is not invariably superior in OF samples; for IAV, nasal swabs remain the preferred method for VI and individual diagnosis. Consequently, OF should be regarded as a tool for group‐level surveillance, whereas serum, nasal swabs, tracheal swabs, faeces or tissue samples are still important for early detection, individual diagnosis, low‐prevalence herds or confirmatory testing.

## 6. Advantages of OF‐Based Diagnostics

OF collection provides a noninvasive alternative to blood sampling, improves animal welfare and is particularly advantageous in swine owing to its simplicity and safety. For pigs, OF samples were collected using cotton ropes or sponges (pen‐based or individual sampling). It minimises stress and injury risk compared with venipuncture, which is often challenging in pigs due to their substantial subcutaneous fat layer. It may therefore reduce sampling‐induced confounding of stress biomarkers such as cortisol and sympathetic markers (e.g., CgA and salivary α‐amylase) [5, 6]. In large‐scale commercial production systems, where individual sampling is labour‐intensive, time‐consuming and impractical, this approach enables efficient group‐level sampling for continuous mass herd surveillance and longitudinal repeated health monitoring [31]. From an economic standpoint, OF sampling is cost‐effective as a single pen‐based sample can represent multiple pigs. This method reduces labour, veterinary involvement, sampling time, animal restraint and stress compared to individual serum sampling. OF samples can be easily collected by farm staff with minimal training by leveraging pigs′ natural chewing behaviour. This approach helps maintain biosecurity by limiting external personnel access to the farm [16]. OF sampling can show comparable sensitivity and specificity performance to conventional biological samples for selected pathogens and biomarkers, particularly when assays are properly optimised and validated for the OF matrix [5, 7]. OF‐based sampling may also facilitate molecular characterisation and epidemiological monitoring of viruses such as IAV, PRRSV, PCV2 and PCV3, particularly when viral load and nucleic acid quality are sufficient [27, 28, 56, 57]. It is also possible to discover novel biomarkers through proteomic studies [14, 16].

## 7. Limitations of OF‐Based Diagnostics

OF diagnostics offer distinct benefits but also have significant drawbacks. Compared with other biological fluids, particularly in serum, biomarkers, antibodies and nucleic acids have lower concentrations in OF because only a limited fraction is transferred from the bloodstream, thereby compromising diagnostic sensitivity [21]. Biomarker stability during shipment requires proper cold storage (≤ 4°C or freezing), as inadequate handling can degrade biomarkers and increase the risk of false‐negative findings [1]. Sample collection can also be affected by several factors, notably pig age and behaviour, housing conditions, pen size and the number of ropes deployed in the pen during sampling. In large commercial pens with a handful of ropes, limited engagement with cotton ropes could affect sample representativeness [14, 45]. Poor participation and dominance‐related rope chewing may diminish diagnostic sensitivity, particularly in pens with low prevalence or when only a limited number of pigs are shedding pathogens. In addition, most diagnostic assays have been developed for blood samples; therefore, adapting them for OF requires methodological adjustments. Interpretation may also be challenging owing to the dearth of well‐established OF‐specific reference ranges for many biomarkers [14]. The presence of environmental contaminants and feed particles in OF may compromise assay performance and elevate the likelihood of false‐positive results, often necessitating additional purification or centrifugation prior to testing [2]. Under field conditions, pigs are often exposed to multiple concurrent infections, which may cause fluctuating antibody levels in OF and complicate detection, especially in herds with low infection rates [58]. Thus, pen‐based OF should be primarily considered a method for monitoring at the group level rather than a substitute for diagnosing individual animals.

## 8. Future Directions in Pig OF‐Based Disease Surveillance

**FIGURE 4:**
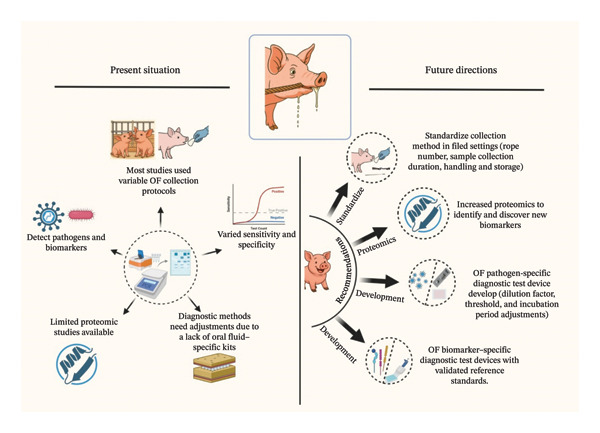
Future directions for OF‐based diagnostics and surveillance in swine.

OF‐based diagnostics are intriguing; still, to facilitate their widespread use in swine health monitoring, OF‐based disease surveillance should focus on improving diagnostic efficiency through refinements to existing approaches (Figure 4). Initially, it is imperative to establish standardised protocols for OF collection to ensure accurate, consistent diagnostics across all production systems, whether in large commercial or free‐ranging farms. This process involves optimising sampling time to increase animal participation in field studies and adjusting the number of ropes on large commercial farms with higher pig numbers per pen [41, 59]. In commercial swine, OF samples both before and after transportation could be an effective way to assess shedding of respiratory pathogens and the stress responses associated with transport. The instability of biomarkers in OF samples underscores the pressing need for effective management of sampling protocols to assure integrity throughout the collection, handling, shipment and storage processes [19, 58, 60]. Ongoing research and standardised optimisation of existing serum‐based assays for OF remain essential until fully validated OF‐specific diagnostic kits are developed. The prioritisation of developing pathogen‐specific OF assays, including OF‐specific ELISA kits and on‐farm point‐of‐care tests, is essential. This development should be accompanied by matrix‐specific validation and regulatory approval. The validation process must evaluate diagnostic sensitivity and specificity, analytical performance, reproducibility, cut‐off optimisation, sample stability and the effects of PCR inhibition, dilution and contamination. These assessments are crucial before employing these assays for official surveillance, disease‐free certification or the detection of economically significant diseases [14]. Future OF‐based studies should incorporate pathogen genotyping, sequence analysis and molecular epidemiology to better understand pathogen diversity, transmission routes and outbreak dynamics in swine populations [28, 56]. Future research on OF surveillance should incorporate a cost–benefit analysis that accounts for labour costs, veterinary expenses, sampling duration, animal restraint and animal stress associated with serum sampling. Additionally, it should consider costs associated with collection materials, cold‐chain maintenance, matrix‐specific assay optimisation and validation, repeat sampling, confirmatory serum testing and follow‐up investigations for false‐positive or false‐negative results. Future research should aim to establish validated OF‐specific reference intervals for key biomarkers, thereby supporting consistent clinical interpretation and diagnostic decision‐making [8, 9, 49]. Advanced OF‐based proteomics is warranted for the identification of novel biomarkers, particularly those indicative of low‐abundance infection or physiological stress, contributing to earlier intervention and more precise health assessment [17, 41]. While this review primarily addresses swine, diagnostic approaches based on OF may also apply to other livestock species. However, their practical implementation necessitates validation specific to each species.

## 9. Conclusions

OF sampling offers a convenient, noninvasive, animal‐friendly approach for monitoring herd health in pigs, especially for detecting infectious pathogens and biomarkers through repeated pen‐level sampling, which supports effective surveillance and molecular monitoring of pathogen shedding. However, although OF is valuable for group‐level surveillance, serum, nasal swabs, tracheal swabs, faecal samples or tissue samples will still be required for early detection, individual diagnosis and confirmatory testing in low‐prevalence herds. This is because lower analyte concentrations, variability in sample quality and representativeness due to pig participation and contamination with feed or environmental material, matrix effects, analyte instability during transport and storage and the dearth of validated assays for swine OF limit its broader deployment. In this review, we scrutinised the shortcomings of OF‐based diagnostics and their current use in the detection of pathogens and biomarkers, focusing on the details of sample collection, handling protocols and method modifications enacted by researchers to improve assay performance. In the future, routine deployment of OF diagnostics will require standardised sample collection and handling techniques, OF‐validated assays with defined threshold values, exact reference ranges for clinically significant biomarkers, field validation, regulatory acceptance and the development of pathogen and biomarker‐specific OF test kits to improve diagnostic reliability. With these developments, OF‐based diagnostics can achieve sustained adoption in herd health monitoring and epidemiological disease surveillance.

## Author Contributions

Conceptualisation: Chandra Shaker Chouhan, Jasim Muhammad Uddin and Sam Abraham; methodology: Chandra Shaker Chouhan and Jasim Muhammad Uddin; data curation: Chandra Shaker Chouhan; writing–original draft preparation: Chandra Shaker Chouhan; writing–review and editing: Jasim Muhammad Uddin, Flaminia Coiacetto and Sam Abraham; visualisation: Chandra Shaker Chouhan; supervision: Jasim Muhammad Uddin, Flaminia Coiacetto and Sam Abraham.

## Funding

The research did not receive financial support from any funding agencies in the public, commercial or nonprofit sectors. Open access publishing facilitated by Murdoch University, as part of the Wiley ‐ Murdoch University agreement via the Council of Australasian University Librarians.

## Disclosure

All authors have read and agreed to the published version of the manuscript.

## Conflicts of Interest

The authors declare no conflicts of interest.
